# Microsatellite-based bottleneck analysis and migration events among four native Turkish goat breeds

**DOI:** 10.5194/aab-67-353-2024

**Published:** 2024-07-17

**Authors:** Eymen Demir

**Affiliations:** Department of Animal Science, Faculty of Agriculture, Akdeniz University, Antalya, 07058, Türkiye

## Abstract

Molecular data analyzed by accurate statistical approaches not only have the potential to investigate demographic events faced in the past, e.g., migration, but they also offer significant hints such as bottleneck effects to take suitable measures for sustainable breeding in farm animals. In this study, the genetic bottleneck and migration events among four native Turkish goat populations, i.e., Hair, Honamlı, Kabakulak, and Norduz, were assessed using 367 multi-allelic data belonging to 28 microsatellite loci. The null hypothesis was not rejected for the Wilcoxon sign rank test in the infinite allele model, two-phase model, and stepwise mutation model, while a normal L-shaped distribution of allele frequencies was observed in terms of mode-shift indicators in four native Turkish goat populations. Both the Wilcoxon sign rank test and the mode-shift indicator demonstrated that Anatolian goat populations have had a lack of recent genetic bottlenecks and have maintained their effective population sizes over the generations. Moreover, the 95 % confidence interval confirmed that the effective population sizes of Hair, Honamlı, and Kabakulak may reach infinity, while the highest effective population size for Norduz was estimated at 794.5, when the lowest allele frequency was considered to be 0.01. Up to four migration events revealed a significant migration from Norduz to Hair and Kabakulak populations. In contrast, no migration from other populations to Norduz was observed, most probably due to its geographic isolation. The bottleneck results may serve as a guide for future management practices, whereas further studies, especially on a whole-genome basis, are needed to confirm migration events among Anatolian goat breeds.

## 1 Introduction

Small ruminants such as sheep and goats are raised in almost all parts of the world due to their multipurpose yields and higher adaptability to harsh environments (Sejian et al., 2019; Dubeuf et al., 2023). Today, meat and milk are essential for a healthy diet in human nutrition, while various products derived from small ruminants (mutton, wool, fleece, skin, manure, etc.) are processed by the textile and agriculture sectors. In rural areas of Türkiye, goat breeding significantly contributes to breeders' incomes (Daskiran et al., 2018), but the country's green areas are also utilized efficiently via grassland-based breeding systems. As highlighted by Şen et al. (2021), approximately 11 million goats belonging to 13 different breeds and varieties are raised in the country, while goat breeding is mainly centered on Hair goats (HAI), which constitute more than 90 % of the total goat population (Karsli et al., 2020). The HAI breed conserves some varieties such as Çandır, Pavga, and Kabakulak (KBK) that were reported to be adapted to different climatic conditions (Karsli et al., 2020) and to possess differences in terms of body size, fertility, and yield traits (Erduran and Kırbas, 2010). The population sizes of other native Turkish goat breeds are predicted to be comparatively low, in which Honamlı(HNM) is raised across the Taurus Mountains of the Mediterranean region including Antalya, Isparta, and Konya provinces, while Norduz (NRD) is reared in a limited region of the country (Van Province) (Daskiran et al., 2018).

Numerous molecular markers have been developed to detect allele frequencies across different loci in which microsatellites allow for detection of the alleles of repeated regions. Since microsatellites are highly polymorphic, they were utilized to reveal genetic diversity and population structure in small ruminants (Karsli et al., 2020; Loukovitis et al., 2023). In parallel with molecular genotyping techniques, accurate statistical methods have been modeled to broaden the use of the same genotypic data for various approaches. Of these approaches, the bottleneck has enabled scientists to investigate recent reductions in effective population size via different statistical models, i.e., the infinite allele model (IAM), two-phase model (TPM), and stepwise mutation model (SMM) (Cornuet and Luikart, 1996). The algorithm compares expected heterozygosities at Hardy–Weinberg and mutation–drift equilibria in the same individual (Koul et al., 2020). Bottleneck analysis may reveal significant information about local goat breeds, as it does not require any information about the breeding history and effective size of the populations. As Kumar et al. (2007) highlighted, the effects of recent bottlenecks could be investigated via genotypic data of 5–20 polymorphic microsatellite loci belonging to 20–30 individuals from each population.

On the other hand, the TreeMix approach facilitates the analysis of migration events among different populations via the maximum-likelihood tree algorithm (Pickrell and Pritchard, 2012). Using polymorphic microsatellite and bi-allelic single-nucleotide polymorphism (SNP) data, TreeMix benefits from allele frequencies and Gaussian approximations for genetic drift to draw a tree in which possible migration events from one population to another are visualized (Flesch et al., 2020). Numerous studies have used the TreeMix algorithm to screen migration events in different livestock species, e.g., cattle (Demir et al., 2023), sheep (Ceccobelli et al., 2023), and goats (Paim et al., 2019). In addition, the magnitudes of these migration events from one population to another can be estimated using numerous statistical models. For example, a Bayesian approach explained by Wilson and Rannala (2003) is available to estimate rates of recent immigration over the last several generations among different populations. Relying on the Markov chain Monte Carlo method, this algorithm calculates the estimation of posterior probabilities to reveal migration rates via different genetic data such as microsatellites and SNPs.

Among native Turkish goats, HAI has the highest effective population size, while HNM, KBK, and NRD are facing a declining trend in terms of census. This trend has forced authorities to take precautions for sustainable use of these breeds in long-term production. Today, numerous local breeds belonging to Anatolian small ruminants are subjected to national conservation programs under the breeder's hand (Güngör and Gürer, 2022). It is noteworthy that genetic bottlenecks, the number of effective population sizes, and gene flow should be periodically monitored to shape and expand conservation studies efficiently. Recently, Karsli et al. (2020) comprehensively assessed the genetic diversity and population structure of four native goats (HAI, HNM, KBK, and NRD) via 255 different alleles detected by 20 microsatellite markers, while bottleneck analysis and migration events were neglected. Hence, by extending the same genotypic data, this study aims to evaluate recent bottlenecks and migration events among four native Turkish goat populations.

## 2 Materials and methods

### 2.1 Animal sampling and molecular genotyping

**Figure 1:**
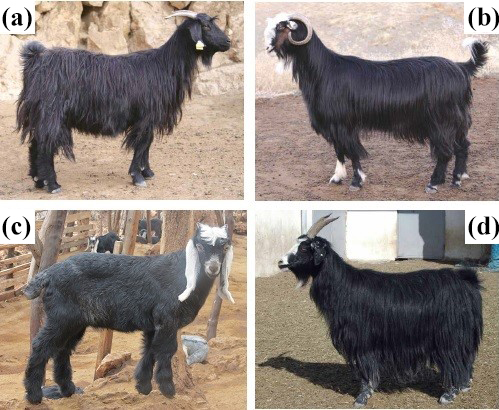
Images of animals belonging to the **(a)** HAI, **(b)** HNM, **(c)** KBK, and **(d)** NRD breeds.

As reported by Karsli et al. (2020), a total of 141 blood samples from both sexes (20 males and 121 females) were collected in 2018 after the ethical statement was approved by the Akdeniz University Animal Experiments Ethics Committee, Antalya, Türkiye (certificate no. 2016.12.01). HAI ( n=60 ), HNM ( n=3 0), and KBK ( n=30 ) were sampled from representative herds raised in Antalya, whereas blood samples of NRD ( n=21 ) were obtained from representative herds raised in Van Province. In order to choose animals that were as pure as possible, breed-specific morphological traits were considered, while oral interviews were conducted with breeders to select unrelated animals. Images of representative animals giving clues in terms of morphological traits per breed are illustrated in Fig. 1.

A salting-out method described by Miller et al. (1988) was utilized to extract DNA from whole blood, which was further genotyped in terms of 20 microsatellite markers (*ILTS005, ILTS011, ILTS087, OarFCB48, SRCRSP03, SRCRSP05, SRCRSP07, SRCRSP09, SRCRSP15, INRA063, INRABERN172, TCRVB6, MAF65, MAF70, ETH10, P19* (*DYA*), *MCM527, CRSD247, OarAE54*, and *SPS113*) recommended by the Food and Agriculture Organization of the United Nations (FAO, 2011). In this study, however, the same animals were also genotyped in terms of an additional eight FAO-recommended microsatellite loci (*BM1818, BM1329, DRBP1, ETH10, INRA0132, OarFCB20, OarAE54, *and *TCRVB6*) by using optimized PCR (50 ng µ L ${}^{-1}$ template DNA, 75 mM µ L ${}^{-1}$ buffer solution, 2.5 mM µ L ${}^{-1}$ deoxynucleotide triphosphates (dNTPs), 10 pmol µ L ${}^{-1}$ of each primer, 5 U µ L ${}^{-1}$ Taq DNA polymerase, and distilled deionized water) and conditions (initial denaturation at 94 °C for 5 min, followed by 30 cycles of denaturation at 94 °C for 30 s, annealing at 50–60 °C for 30 s, and extension at 72 °C for 30 s with the final extension at 72 °C for 10 min). Eight microsatellite loci were amplified separately under the same laboratory conditions (molecular genetic laboratory of the Department of Animal Science, Akdeniz University, Antalya Province of Türkiye) supplied with a 96-well device (ARKTIK Thermal Cycler, Thermo Scientific, USA). Fragment analysis was carried out using a 96 automated capillary electrophoresis system (Advanced Analytical Technologies, Iowa, USA), while band sizes were confirmed using PROSize^®^ 2.0 version 1.3.1.1 (Advanced Analytical Technologies, Iowa, USA). The dataset reported by Karsli et al. (2020) was merged with the current study to obtain final data containing 367 different alleles from 28 microsatellite loci for statistical analyses.

### 2.2 Statistical analysis

Two different approaches were taken using Bottleneck v1.1.2.02 (Piry et al., 1999) to test whether the populations have recently faced a significant reduction in the effective population size. Being the qualitative approach, heterozygosity-excess-based statistics of the Wilcoxon sign rank test were calculated in three models, i.e., the IAM, SMM, and TPM. Additionally, the mode-shift indicator test, which is based on the allele frequency distribution, was used as a qualitative approach to reveal bottleneck effects in four goat populations. The results of the mode-shift approach were visualized by the plot function implemented in the R environment (https://www.r-project.org, last access: 15 July 2024). In order to validate the results of the bottleneck analysis, the linkage disequilibrium-based effective population size ( $N_{e}$ ) was estimated using the allele correlation between the loci. To do so, the LDNe software (Waples and Do, 2008) was run with default parameters to detect the effective population size per breed together with the 95 % confidence interval for lower and upper values.

Four native goat populations were screened using a tree-based approach (TreeMix) in order to reconstruct historical relationships among them such as population splits and gene flow. The mean and variance in length at each microsatellite locus were computed per population to create an input file for the TreeMix software (Pickrell and Pritchard, 2012) that was further tested up to all possible migration events in 20 iterations per edge with 1000 bootstrap values. In order to visualize the trees, the outputs recovered from the TreeMix software were processed by the plot_tree function of the BITE package (Milanesi et al., 2017) implemented in the R environment (https://www.r-project.org). Further, the migration rates for each event were calculated using the Bayesass v1.3 software (Wilson and Rannala, 2003) with 1000 iterations, the results of which were visualized by the circlize package (Gu et al., 2014) implemented in the R environment (https://www.r-project.org).

## 3 Results

In this study, three mutation–drift equilibrium-based mutation models combined with the Wilcoxon sign rank test were utilized to determine whether the effective population sizes have been sustained or reduced in the recent past among four native Turkish goat populations. The significance of one tail for heterozygosity excess in the Wilcoxon sign rank test across the studied goat populations is summarized in Table 1. Probability values for all native Turkish goat populations indicated that the null hypothesis ( P<0.05 ) should be accepted for all the mutation models (IAM, TPM, and SSM). Based on this criterion, it could be stated that none of these goat populations has experienced recent bottleneck effects.

**Table 1:** Probability values for bottleneck analysis in a Wilcoxon sign rank test in four native Turkish goat populations in three mutation models.

	IAM	TPM	SMM
HAI	1.00000	0.99998	0.76255
HNM	1.00000	0.99992	0.42041
KBK	1.00000	0.99721	0.76255
NRD	0.99884	0.97578	0.73916

HAI: Hair goat; HNM: Honamlı; KBK: Kabakulak; NRD: Norduz; IAM: infinite allele model; SMM: stepwise mutation model; TPM: two-phase model.

**Figure 2:**
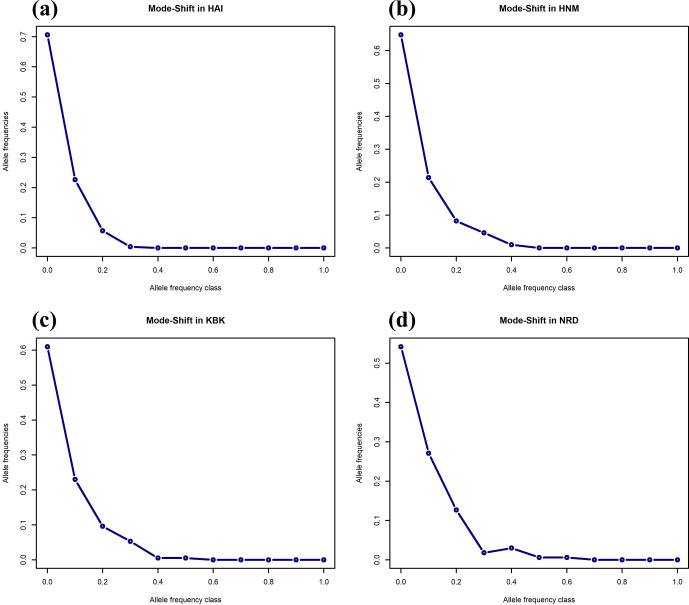
Mode-shift test for the bottleneck in four native Turkish goat populations (HAI, HNM, KBK, and NRD).

As an alternative to the Wilcoxon sign rank test, the mode-shift algorithm, a qualitative approach based on the distribution of allele frequencies, was also performed to assess the reduction in the effective population sizes of the studied goat populations (Fig. 2). A normal L-shaped distribution of allele frequencies was observed in four native Turkish goat populations, most probably due to the lack of recent bottleneck effects (Fig. 2).

On the other hand, the effective population size was also estimated using the linkage disequilibrium model for each population, the results of which are given in Table 2. No matter what the lowest number of the allele frequency is, negative $N_{e}$ values were detected in the HAI and HNM breeds, while positive $N_{e}$ values ranging from 391.3 (NRD; 0.02) to 14427.3 (KBK; 0.01) were observed between the KBK and NRD populations (Table 2). The upper values for the 95 % confidence interval revealed that HAI, HNM, and KBK have infinite population sizes, whereas the effective population size of NRD varied between 524.8 and 794.5 based on the lowest allele frequency (Table 2).

**Table 2:** Estimates of LDNe-based effective population size with a 95 % confidence interval in four goat populations.

Population	LAF	$N_{e}$	Lower $N_{e}$	Upper $N_{e}$
	0.05	-1336.2	203.1	Infinite
HAI	0.02	-1401.0	203.8	Infinite
	0.01	-1401.0	203.8	Infinite
	0.05	-102.9	-200.5	Infinite
HNM	0.02	-109.6	-188.1	Infinite
	0.01	-106.6	-171.5	Infinite
	0.05	2106.2	197.7	Infinite
KBK	0.02	-2114.2	311.4	Infinite
	0.01	14427.3	294.9	Infinite
	0.05	391.3	308.9	524.8
NRD	0.02	537.3	430.1	708.1
	0.01	606.4	487.5	794.5

HAI: Hair goat; HNM: Honamlı; KBK: Kabakulak; NRD: Norduz; LAF: lowest allele frequency; $N_{e}$ : effective population size.

**Figure 3:**
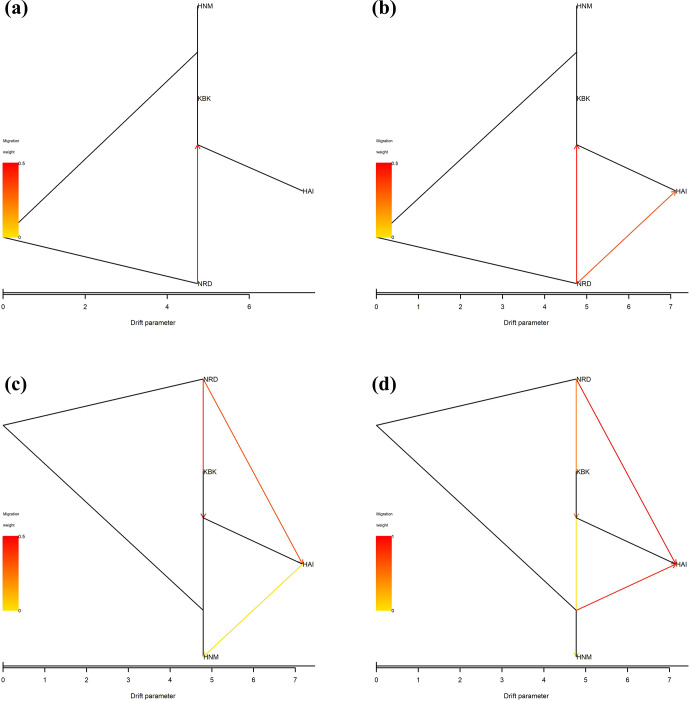
Maximum-likelihood tree with **(a)** one, **(b)** two, **(c)** three, and **(d)** four migration events for four native Turkish goat populations (HAI, HNM, KBK, and NRD).

**Figure 4:**
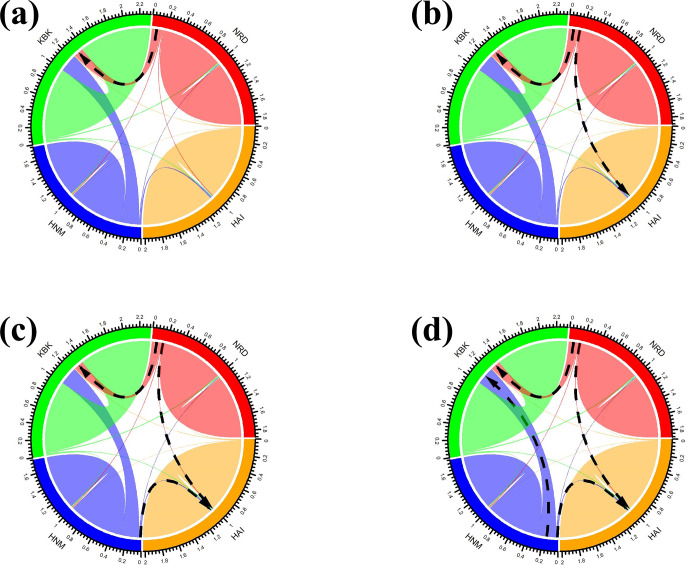
Migration rates among native Turkish goat breeds based on **(a)** one, **(b)** two, **(c)** three, and **(d)** four migration events. The black arrows indicate the directions of the most intense migration rates (HAI, 140 HNM, KBK, and NRD).

In this study, all possible migration events aimed to assess population splits and gene flow in which a maximum of four migration events were visualized using the TreeMix algorithm. Therefore, a total of four scenarios were taken into consideration to assess migration events among four native Turkish goat populations. Allowing only one migration event showed that there was a migration from NRD to KBK (Fig. 3a) with a 0.126 migration rate (Fig. 4a). The assumption of two migration events drew a second migration edge from NRD to HAI (Fig. 3b) with a lower value (0.014) of the migration rate (Fig. 4b). The third scenario showed that there was also a migration from HAI to HNM (Fig. 3c) with a migration rate of 0.070 (Fig. 4c). Under the assumption of four migration events, an edge from HNM to HAI (Fig. 3d) was drawn with a value of 0.130 for the migration rate (Fig. 4d).

## 4 Discussion

Although three different tests known as the sign, standardized difference, and Wilcoxon sign rank in three different mutation models (IAM, TPM, and SSM) are available to analyze bottleneck effects in livestock species, many studies have revealed that these approaches may show inconsistent results. For example, a study utilizing a total of 23 microsatellite loci and benefitting from the sign, standardized difference, and Wilcoxon sign rank tests discovered a genetic bottleneck in two goat breeds (Bidri and Nandidurga) reared in India in terms of the SMM and TPM, while these populations were in mutation–drift equilibrium using the IAM (Tantia et al., 2018). Another study based on 25 microsatellite markers revealed that Konkan Kanyal, an indigenous goat breed reared in India, has experienced a recent genetic bottleneck according to the standardized difference test in the TPM and SMM, whereas mutation–drift equilibrium was reported regarding the sign and Wilcoxon sign rank tests in three possible mutation models (Mishra et al., 2012). These inconsistencies among statistical tests and mutation models have been taken into consideration by several previous studies (Ağaoğlu and Ertuğrul, 2012; Peery et al., 2012; Selepe et al., 2018; Akay et al., 2020; Kabasakal, 2023), suggesting that the Wilcoxon sign rank test in the TPM and a mode-shift indicator give more precise results for genetic bottleneck analysis. Therefore, in this study, the Wilcoxon sign rank test and the mode-shift indicator were preferred to assess the effects of the genetic bottleneck in four native Turkish goat populations. Neither the quantitative (Wilcoxon sign rank test) nor qualitative (mode-shift indicator) algorithms showed any effect of the genetic bottleneck on Anatolian goat populations, suggesting that an effective population size has been maintained over generations.

These findings were also consistent with previous studies focusing on the determination of the genetic bottleneck in some native small ruminant breeds reared in Türkiye. For example, using the Wilcoxon sign rank test with the TPM, Ağaoğlu and Ertuğrul (2012) reported a lack of a genetic bottleneck in five Anatolian goat breeds (Angora, Kilis, HAI, HNM, and NRD) genotyped by 20 microsatellite loci. Akay et al. (2020) highlighted that even the endangered Güney Karaman sheep breed, which was genotyped by 16 microsatellite loci, has sustained the effective population size over generations using the Wilcoxon sign rank test with the TPM. Similarly, Kabasakal (2023) genotyped the Karacabey Merino sheep breed with a total of 14 microsatellite markers in which no genetic bottleneck was reported using the Wilcoxon sign rank test with the TPM.

The results of the bottleneck analysis were also consistent with the estimated effective population size in which the negative $N_{e}$ values were observed in HAI and HNM in all the scenarios related to the lowest allele frequency. A similarly negative $N_{e}$ value was observed in the KBK population in case the lowest allele frequency was 0.02. A clear explanation for negative $N_{e}$ values was given by Do et al. (2014), indicating that $N_{e}$ estimation is based on two genetic components such as genetic drift and sampling of a finite number of individuals. However, the actual amount of the sampling error can be greater than the estimated value, which results in a negative value of $N_{e}$ together with the “infinite” upper confidence interval value. In this case, Do et al. (2014) indicated that, due to sampling error, there is no evidence of variation in the genetic characteristics caused by a finite number of parents. In addition, Peel et al. (2013) reported that negative $N_{e}$ values could be interpreted as a sign of no evidence of genetic drift. Indeed, the upper value for the 95 % confidence interval indicated that the effective population sizes of HAI, HNM, and KBK were infinite. In contrast, the highest effective population size of NRD was estimated to be 794.5.

This result is consistent with the breeding history of the breed since NRD survives as small herds in a limited region of the country. This finding is crucial for national conservation programs because, as highlighted by Alderson (2009), breeds with 500–1500 breeding females are categorized as “vulnerable”. Therefore, a comprehensive conservation study is to be initiated to increase the effective population size of the NRD breed. Otherwise, there is a significant risk of NRD being classified as “endangered” if the number of active breeding females becomes lower than 500 individuals.

In this study, migration of up to four events was allowed to assess population splits and gene flow. In all the scenarios, migration from NRD to the other populations (KBK and HAI in particular) was observed, whereas no migration edge was drawn from other populations to the NRD breed. It is normal to observe migration among HAI, HNM, and KBK because they were sampled from the same province (Antalya). Even though NRD has been geographically isolated from other goat populations for decades, HAI, HNM, and KBK still conserve gene flow from the NRD breed. In addition to all the scenarios of migration events, NRD was found to be genetically different from other populations. Although no previous study is available in the literature to assess migration events among Anatolian goat breeds via microsatellite data, population differentiation was reported via Bayesian and tree-based algorithms in several studies (Ağaoğlu and Ertuğrul, 2012; Gül et al., 2020). Karsli et al. (2020) highlighted for the first time that NRD was genetically different from HAI, HNM, and KBK. The results of the present study show similarities to the findings of Karsli et al. (2020). This is not surprising, since both studies have benefitted from the same sampling strategy. Therefore, more studies are required to obtain deeper knowledge of migration events among Anatolian goat breeds. Indeed, Karsli et al. (2020) also highlighted that, rather than microsatellite loci, denser genetic data obtained from SNP array and next-generation sequencing technologies are required to investigate past events related to phylogeny in native Turkish goat breeds.

## 5 Conclusions

In this study, the presence of the genetic bottleneck was assessed via the Wilcoxon sign rank test and the mode-shift indicator in four native Turkish goat populations genotyped by 28 microsatellite loci. Neither approach demonstrated any sign of a genetic bottleneck, suggesting that these populations have maintained their effective population size over generations. However, except for HAI, these goat populations are in danger of extinction due to the fact that their population size tends to decrease year by year. Special attention should be paid to the NRD breed, which has the lowest effective population size. Moreover, the effects of bottlenecks as well as effective population size should be monitored periodically to take effective management measures. Migration of up to four events revealed migration from NRD to other populations, whereas no migration from other populations to NRD was detected, most probably due to geographic isolation. Maintaining this kind of isolation may result in decreased genetic variability as well as bottleneck effects in the future. Therefore, it is highly recommended that the effective population size of NRD should be conserved, while their geographic distributions could be extended to nearby zones.

## Data Availability

The datasets used in this study for eight microsatellite markers (*BM1818, BM1329, DRBP1, ETH10, INRA0132, OarFCB20, OarAE54,* and *TCRVB6*) are available upon request via a Material Transfer Agreement signed by the corresponding author for scientific purposes only.
